# Antioxidant, antibacterial, antitumor, antifungal, antiviral, anti-inflammatory, and nevro-protective activity of *Ganoderma lucidum*: An overview

**DOI:** 10.3389/fphar.2022.934982

**Published:** 2022-07-22

**Authors:** Darija Cör Andrejč, Željko Knez, Maša Knez Marevci

**Affiliations:** ^1^ Faculty of Chemistry and Chemical Engineering, Maribor, Slovenia; ^2^ Laboratory Faculty of Medicine, Maribor, Slovenia

**Keywords:** *Ganoderma lucidum*, bioactive function, active compounds, polysaccharides, triterpenoids

## Abstract

*Ganoderma lucidum* is a very medicinal mushroom that has been utilized in Oriental medicine for many years. It has a wide range of pharmacological and therapeutic properties, and has been used for many years as a health promoter. It contains various biologically active compounds that improve the immune system and have antioxidant, antitumor, anti-inflammatory, antifungal, and antimicrobial properties. Active compounds include triterpenoids and polysaccharides, as well as proteins, lipids, phenolics, sterols, etc. In the following review, we summarize briefly their biological activities, such as antioxidant, anti-bacterial, anti-fungal, antitumor, anti-viral, and anti-inflammatory activity. Although *Ganoderma* has a number of medicinal effects that have been confirmed by the *in vitro* and *in vivo* studies summarised in this review, there are some limitations. Clinical trials face mainly a lack of pure constituents. Accurate identification of the compounds obtained is also problematic. In addition, most of the included studies were small, and there were concerns about the methodological quality of each study. Studies have shown that Ganoderma has valuable potential for the prevention and treatment of cancer. In any case, *G. lucidum* cannot be used as first-line therapy for cancer.

## Introduction

Nutraceuticals, or functional foods, contain bioactive compounds isolated from natural tissues that promote a wide range of activities in addition to their nutritional value. Extracts from medicinal mushrooms are used traditionally in traditional Chinese medicine for many years. The fruiting body, spores and mycelium of medicinal mushrooms contain many bioactive components, from which the medicinal properties are derived (Seweryn et al., 2021). One of these mushrooms is *Ganoderma lucidum,* which was discovered over 2,400 years ago, and is known to promote health, longevity, and mental growth. It is also recognized as a powerful immune booster, providing strong protection for the whole body (Wang J. et al., 2014). The substances found in the mushroom include triterpenoids, polysaccharides, nucleotides, sterols, steroids, fatty acids and proteins/peptides, and have numerous medicinal effects (Sanodiya et al., 2009; Sindhu et al., 2021). The main pharmacologically active compounds from *G. lucidum* are triterpenoids and polysaccharides. It has been found out that its bioactive compounds indicate antimicrobial (Karwa and Rai, 2012), antitumor (Sliva, 2003; Zhang et al., 2003; Kao et al., 2013), anti-inflammatory (Hasnat et al., 2015; Wen et al., 2022), hypolipidemic (Chen et al., 2005), antiatherosclerotic (Lai et al., 2020), anti-fungal, and anti-viral activity (Akbar and Yam, 2011; AL-jumaili et al., 2020; Cheng et al., 2021).

**FIGURE 1:**
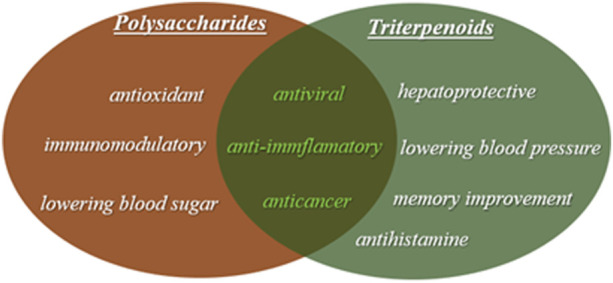
Bioactive activities of triterpenoids and polysaccharides.

Several years of studies have confirmed that *G. lucidum* is an immunostimulant and a strong antioxidant. It can now be used as an adjunct to prevent the effects of chemotherapy and to tackle cancer (Jin et al., 2012). The different bioactive activities of triterpenoids and polysaccharides are shown in Figure 1. The polysaccharides of (GLPs) have many biological activities like: immunomodulatory, antineurodegenerative, antidiabetic, anti-inflammatory, anticancer, and antibacterial. In particular, *β*-d-glucans are well known to have biological and physiological activities (Wang X. M. et al., 2014).

Triterpenoids possess antihypertensive, hypocholesterolemic, hepatoprotective and antihistaminic effects, as well as antitumor and antiangiogenic activity. Over a hundred of triterpenoid compounds have been found in extracts of *G. lucidum*. They are divided into *Ganoderma* acids (GA) or *Ganoderma* alcohols (GAlc). Various triterpenoids contain large amounts of lucidenoic acids (Bharadwaj et al., 2019).

## Polysaccharides and triterpenoids from *G. lucidum*

Many natural biological polysaccharides have been shown to be good antioxidants. These include the polysaccharides of *G. lucidum* (GLPs). To date, a number of polysaccharides have been isolated from *G. lucidum*. The most abundant of these are β-D-glucans, α-D-glucans, α-D-mannans and polysaccharide-protein complexes. The type of extraction method depends on the structure of the cell wall, although hot water is the most frequently used media for extraction of these compounds (Siwulski et al., 2015).

The polysaccharide chains are stabilized by hydrogen bonds, and can form the tertiary structure of the triple helix. Such tertiary structures of the triple helix have also been confirmed in β-D-glucans of *G. lucidum* (Synytsya and Novák, 2013)*.* The chemical structure of polysaccharides depends on the isolation method and the type of growth medium used. Crude polysaccharides contain impurities like proteins. This limits their use severely in medical and food applications, while proteins in combination with polysaccharides can trigger allergic reactions. For this reason, so-called deproteinization is used to obtain polysaccharides from natural biomass (Zeng et al., 2019).

Pure β-glucans are the main isolated compound from the *G. lucidum* fruiting body. However, heterofucans, heteromannans and their complexes with peptides, are also found (Sohretoglu and Huang, 2018). Low-molecular-weight polysaccharides GLP-1, which is a pure glucose polysaccharide, and GLP-2, which is composed of β-D-glucose and α-D-galactose, have been studied widely (Liu et al., 2010). Many methods are known to be used for the purification of polysaccharides, such as: trichloroacetic acid (TCA) precipitation (Fic et al., 2010), the enzymatic method (Wang et al., 2007, 31461), lead acetate precipitation (Chen et al., 2012), the Sevag method (Qin et al., 2002), salting out (Huang et al., 2012). With the Sevag method, a high proportion of polysaccharides are lost due to the harsh chemical treatment. In the case of lead acetate precipitation, heavy metal contamination is difficult to avoid. Salt precipitation is very often used for protein precipitation. TCA precipitation provides an operationally simple and efficient protein precipitation process. The enzymatic method is also a simple, environmentally friendly, efficient and inexpensive way to purify polysaccharides. Therefore, these three methods are the most used to produce deproteinized GLP. As mentioned above, the antioxidant activity of polysaccharides depends on their structure. The molecular weight, monosaccharide composition and chain conformation of polysaccharide are highly correlated with antioxidant activity (Zeng et al., 2019). During the processing steps, the primary structure of the polysaccharides can be destroyed, thus reducing the yield of the polysaccharides, as well as their antioxidant activity (Zeng et al., 2019).

**FIGURE 2:**
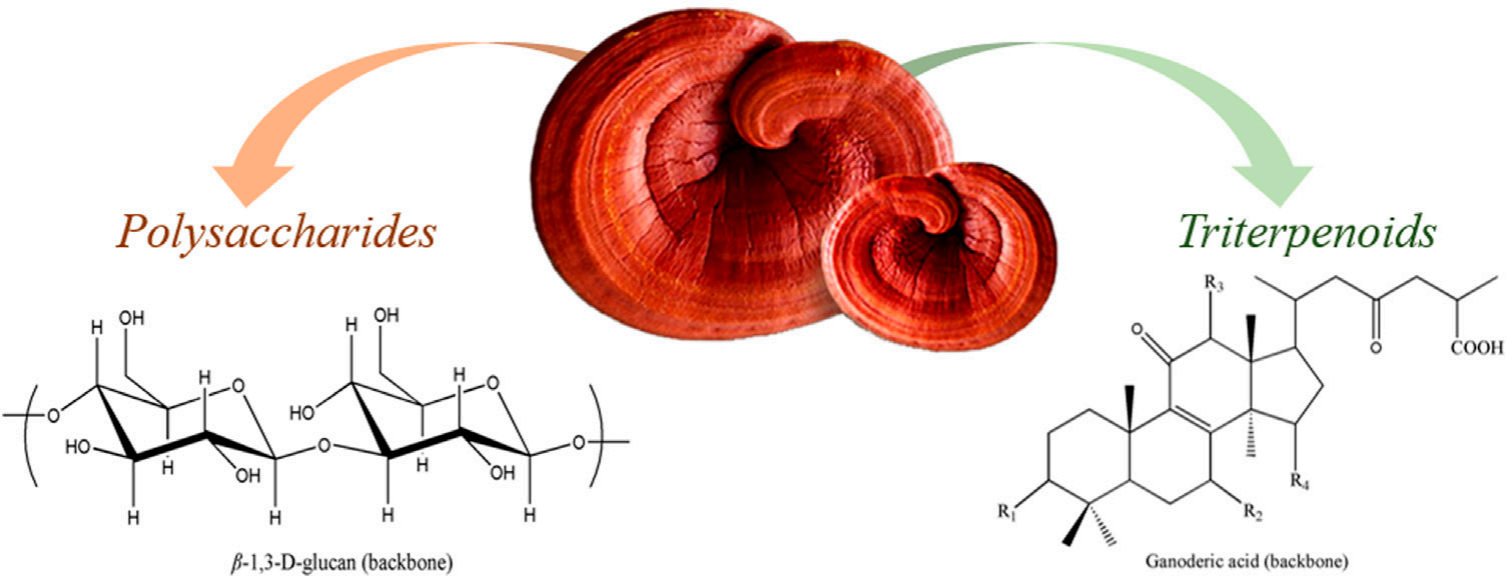
*G. lucidum* β-1,3-glucan and ganoderic acid.

**FIGURE 3:**
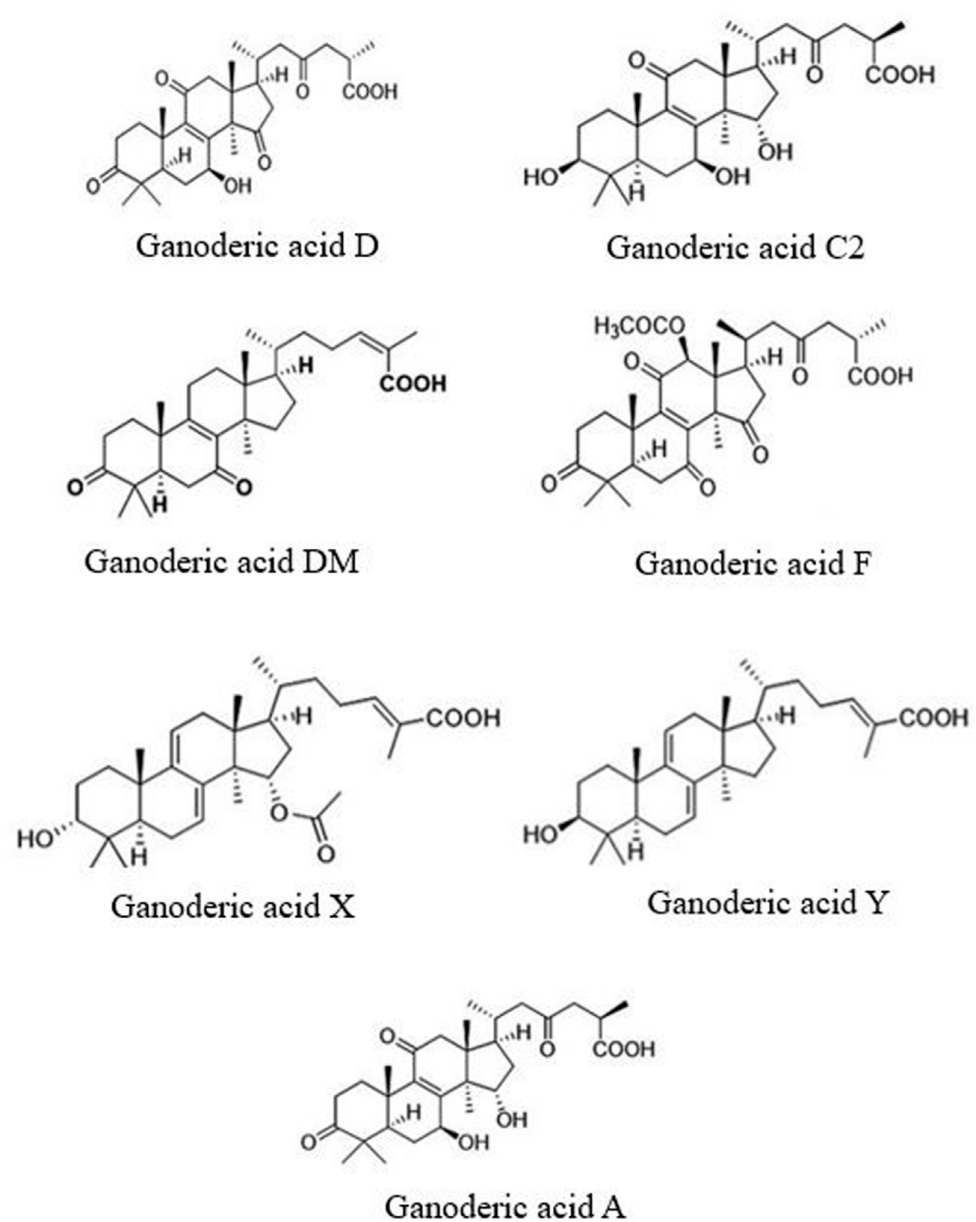
Structural formula of common Ganoderic acids found in *Ganoderma lucidum*.

Besides polysaccharides, *G. lucidum* contains triterpenoids, a subtype of terpenes, which are widely distributed in the plant. Their basic skeleton is C30, with molecular masses between 400 and 600 kDa. Their chemical structure is complex and highly oxidized. As they are pharmaceutically active compounds, they contribute to the biological capacity of *G. lucidum*. A large variety of triterpenoids, mostly ganoderic acids (GAs) (Yuen and Gohel, 2005; Shi et al., 2010), have been shown to be involved in several biological effects, including anti-inflammatory (Akihisa et al., 2007), anti-tumor (Li et al., 2013), anti-HIV (El-Mekkawy et al., 1998) and hypolipidemic activities. Ganoderic acids show an amphipathic effect on platelet aggregation. To date, more than a hundred triterpenes have been identified in *G. lucidum* (Yue et al., 2010), which also showed to have a number of enzymatic inhibitory effects (Sharma et al., 2019). Structural formula of β-1,3-glucan and ganoderic acid are presented in Figure 2. Structure of the most common ganoderic acids are presented on Figure 3.

## Biological activities

### Antioxidant activity

Various *in vitro* antioxidant assays confirmed the antioxidant potential of polysaccharides and polysaccharide complexes extracted from *G. lucidum* (Bukhman et al., 2007)*.* Large amounts of free radicals cause a redox imbalance in the body, leading to oxidative damage in the tissues. Damage to proteins, lipids and DNA caused by oxidative stress is a significant factor in the development and progression of diseases. Polysaccharides isolated from *G*. *lucidum* exhibit antioxidant activity and protect tissues from reactive oxygen species toxicity. Moreover, they help sustain the oxidative state of the body (Jeong and Park, 2020).

The molar mass of polysaccharides is very important, because it is also related to the biological properties of the polysaccharide. The number of reductive ends of the hydroxyl group that can absorb and scavenge free radicals is crucial. Due to this, polysaccharides with a low molecular weight can indicate a higher antioxidant capacity (Ning et al., 2003). For example, a polysaccharide with low molecular weight demonstrated higher capacity in scavenging free radicals and chelating Fe(II). The reason for the better activity could be the molar mass (Liu et al., 2010).

There are numerous studies on the antioxidant activity of polysaccharides. Fan et al. reported that one of the factors affecting the antioxidant activity of polysaccharides is drying. They showed that. compared to other types of drying, the samples dried by hot air, vacuum and lyophilization demonstrated stronger antioxidant activity (Fan et al., 2012).

The polysaccharides also showed some antioxidant effect in *in vitro* studies. They were able to increase the activities of antioxidant enzymes (SOD, CAT, and GPx). They lower the levels of IL -1β, IL -6, and TNF-α in rats with cervical cancer (XiaoPing et al., 2009).

Yan et al. (2019) performed extraction of *G. lucidum* polysaccharides using electrolytically oxidized water. Compared with the conventional extraction method, the electrolytically oxidized water is an environmentally friendly and economically achievable technique. Polysaccharides prepared in this way showed strong antioxidant activity.

Kao et al. (2012) obtained β-1,3-glucan of high purity. The study of its biofunctions show actions against H_2_O_2_-induced apoptosis. Consequently, it is able to exert antioxidant effects by inhibiting SMases.

The most abundant GLP is formed from several amino acids. Sugars are connected by β-glycosidic bonds. GLP has been shown to improve non-enzymatic and enzymatic antioxidants, serum insulin levels and lipid peroxidation. In addition, high antioxidant activity has been demonstrated *in vitro* and *in vivo* (Jiang et al., 2012).

Li J. et al. (2020) demonstrated that GLPs increased white blood cell and lymphocyte amounts. Serum levels of IgG and IgA immunoglobulins were also examined, and higher IgA levels were observed.

Xu et al. (2019) investigated natural polysaccharides (GLP) and degraded polysaccharides by the ultrasonic method (GLP_UD_) and evaluated their hypolipidemic and antioxidant activities. GLPs have shown biological activity on superoxide dismutase and glutathione peroxidase. Malondialdehyde levels were heightened in the serum and liver of mice fed a high-fat diet. From this point of view, GLP can be considered as a new agent for the treatment of hyperlipidemia . Adeyi et al. (2021) also reported incredible biological effects, characterized by the *in vitro* and *in vivo* antioxidant activities of GLP extracts.

Biochemical transformations produce free oxygen and nitrogen radicals. These are highly reactive and cause mitochondrial disorders. Oxidative stress promotes the aging process and is associated with many neurodegenerative diseases, metabolic syndromes, and tumors. The antioxidant activity of polysaccharides does not depend on a single factor, but is a combination of several factors. This requires clarification of the structure, confirmation, and mechanisms affecting antioxidant activity. Further studies should focus on evaluating the degree of toxicity and assessing its potential efficacy in clinical trials.

In addition to polysaccharides, the triterpenoids of *G. lucidum* also exhibit antioxidant activity. To determine the antioxidant activity of triterpenoids extracted from G. lucidum, Dong et al. (2019) used the ability to scavenge two radicals, DPPH and ABTS. Wang et al. isolated some triterpenoids. Two of them, Lingzhin E and Lingzhin F, exhibited antioxidant activity by the ABTS method Zheng et al. (2020) used ethanol maceration to extract the triterpenoids and evaluated their antioxidant activity using the DPPH method, which depends on the concentration of terpenoids.

Zhu et al. (1999) mentioned that the fraction with terpenes consisted of ganoderic acids A, B, C, and D, lucidic acid B and ganodermanontriol as the main compounds, and were noted to have the strongest antioxidant effect.

Joseph et al. (2009) performed chloroform extraction of *G. lucidum*. The extract showed important antioxidant activity and lipid peroxidation inhibitory activity. In addition, methanolic extracts of *G. lucidum* were found to stop kidney damage by restoring the antioxidant protection system of the kidneys (Sheena et al., 2003b). All studies suggest that the antioxidant activities of *G. lucidum* may play an important role in inhibiting lipid peroxidation in biological systems.

### Antibacterial activity

Terpenes, lectins, polysaccharides, etc., are considered antimicrobial compounds, and act on the bacterial cytoplasmic membrane (Kalam et al., 2010). The compounds found in *G. lucidum* can inhibit both gram-positive and gram-negative bacteria. For instance, an aqueous extract of *G. lucidum* can inhibit 15 species of gram-positive and gram-negative bacteria.

It has been reported that some compounds, such as ganomycin and triterpenoids, have broad spectrum antibacterial activity (Shah et al., 2014). Culture fluids from *G. lucidum* show antibacterial activity against bacterial plant pathogens (Robles-Hernández et al., 2021).

The extracts from *G. lucidum* were tested using the sulforhodamine B staining method for antiproliferative activity and the microdilution plate method. The results revealed that all five extracts produced an effective zone of inhibition, with the best by a methanol extract against *Escherichia coli* and *Pseudomonas aeruginosa* (Radhika, 2021). Sknepnek et al. (2018) prepared a *G. lucidum* hot water extract which was used for novel, health-promoting kombucha products. The liquid *G. lucidum* beverage showed inhibitory activity against *Staphylococcus epidermidis*, *Rhodococcus equi*, *Bacillus spizizenii, B. cereus,* and *R. equi.*

Moreover, peptides isolated from *G. lucidum* showed increased antibacterial activity against *Escherichia coli* and *Salmonella typhi.* This is probably due to two mechanisms: Inhibitory action and formation of reactive oxygen species, and the induction of intracellular protein leakage in bacterial cells (Mishra et al., 2018). Ergosta-5,7,22-triene-3β,14α-diol (22Z) was isolated from *G. lucidum.* It was proven that the component exhibited significant activity against *Methicillin-Resistance Staphylococcus aureus* (MRSA) and *Streptococcus pyogenes* (Sande et al., 2020).

The researchers investigated various extracts of *G. lucidum*, such as methanolic, chloroform, acetone and aqueous extracts, and observed antibacterial activity against different bacteria, such as *Bacillus subtilis, Staphylococcus aureus, Enterobacter aerogenes, Corynebacterium diphtheria, Escherichia coli, Salmonella* sp.*,* and *Pseudomonas aeruginosa* (Kalam et al., 2010; Singh et al., 2014; Goud et al., 2019; Radhika, 2021)*.* Furthermore, studies by Heleno et al. (2013) showed that some of the *G. lucidum* extracts indicate a higher antimicrobial activity than antibiotics such as streptomycin and penicillin. All these studies suggest that *G. lucidum* inhibits the development of various bacterial diseases.

### Antifungal activity

There are very few publications on the antifungal activity of *G. lucidum.* Wang and Ng (2006) isolated the so-called ganodermin antifungal protein antifungal protein successfully, which inhibits the mycelial growth of *Botrytis cinerea*, *Fusarium oxysporum*, and *Physalospora piricola*.

Methanolic and aqueous extracts were examined against *Penicillium sps*., *Aspergillus Fumigatous, Aspergillus niger, Aspergillus flavus,* and *Mucor indicus.* Strong activity against *Mucor indicus* was observed (Sridhar et al., 2011). In the study by Heleno et al. (2013), an extract from *G. lucidum* exceeded the antifungal activity against *Trichoderma viride*, compared to the activity of known Standards, i.e., bifonazole and ketoconazole. Yang et al. (2022) reported that the combination of *Ganoderma* polysaccharides and small amounts of chemical fungicides was found to be effective in controlling wheat brood, root rot, and corn stalk rot successfully in continuous wheat growing areas.

### Antitumor activity

A tumor needs a constant supply of nutrients to survive. Invasive cancer cells are spread by blood and lymph vessels. Therefore, agents must be used that can inhibit angiogenesis. Metastasis can also be controlled by targeting factors such as cell adhesion, invasion and migration. Chemotherapy inhibits angiogenesis, which means that it reduces the formation of blood vessels that supply the tumor , thereby reducing its nutrient supply (Sharma et al., 2019). Polysaccharides and triterpenes from *G. lucidum* have been shown to have chemopreventive and antitumor activity.

Studies indicate that different extracts or isolated compounds from *G. lucidum* act as a carcinostatic on different cancer cell lines, such as, lung (Li et al., 2013), colon (Li P. et al., 2020), pancreas (Chen et al., 2022), liver (Li et al., 2005), breast (Barbieri et al., 2017; Jiao et al., 2020), skin (Shahid et al., 2022), prostate (Wang et al., 2020). Ganoderic acids (GAs) -Mk, -S, -Mf, -R, -Mc, -T showed activities against the two tumor cell lines metastatic lung tumor cell line 95-D and human cervical cancer cell line HeLa (Li et al., 2013). GA—H and GA—A suppress the growth and invasive behavior of breast cancer cells by modulating AP-1 and NF-κB signaling (Jiang et al., 2008). It is obvious that the ganoderic acids are the compounds that play a key role in the biological activity of triterpenes. Some of the ganoderic acids and lucidenic acids, with their effects on tumor cells, are listed in Table 1.

**TABLE 1:** Triterpenoids with antitumor action on different cell lines.

Triterpenes	Tumor cell	Target	References
Ganoderic acid -Mk, -S, -Mf, -R, Mc, -T, -Me	Lung: 95D, LLC	MMP 2/9	Chen et al. (2008), Chen et al. (2010); Li et al. (2013)
Ganoderic acid -A, -F, -H, -Me	Breast: MDA-MB-231	AP-1, NF-κB, uPA, Cdk4	Jiang et al. (2008); Li et al. (2012)
Ganoderic acid -X, -T, -E, -B	Liver: HuH-7 , SMMC7721, HepG2	ERK, JNK	Li et al. (2005); Tang et al. (2006); Hsu et al. (2008); Weng et al. (2008)
Ganoderic acid -T, -Me	Colon: HCT-116, HCT-8, Ls174t	NFκB-α, MMP-9, uPA, iNOS	Chen et al. (2010); Xu et al. (2010)
Ganoderic acid -D	Cervical: HeLa	AHA1, Cytokeratin 19, Cytokeratin 1, PRDX3	Yue et al. (2008)
Ganoderic acid -DM	Prostate: PC-3, LnCaP	MMP-2 MMP-9	Johnson et al. (2010)
		IL-1, IL-6, TNF-α, CCL-2/MCP-1	
Lucidenic acid -C, -A, -N, -B.	Leukemia: HL 60	Bcl-2, caspase-9, caspase-3	Hsu et al. (2008)

Triterpenes also cause cell cycle arrest. They first downregulate cyclin D1 in the G1 phase of cell growth, and then inhibit PKC activity in the G2 growth phase. They also induce apoptosis in cancer cell lines. Furthermore, they prevent tumor metastasis by modulating MMPs and IL-8, and inhibit the excretion of inflammatory cytokines (Shahid et al., 2022).

On the other hand, there are *Ganoderma* polysaccharides (GLPs) which have the ability to boost the host’s immune response by stimulating macrophages, NK cells and T lymphocytes (Pan et al., 2013). Pan et al. isolated GLPs that enhance the anti-tumor immune response by stimulating the activity of natural killer cells and cytotoxic T lymphocytes (Pan et al., 2013). Sun et al. tested polysaccharides for lymphocyte activation. The polysaccharides were incubated with a tumor cell line that lacked antigen presentation. They showed that polysaccharides can stimulate melanoma cells to proliferate lymphocytes (Sun et al., 2011). β-glucan from *G. lucidum* showed considerable inhibition of the S180 tumor growth in mice *in vivo* (Fu et al., 2019). According to the studies by Wiater et al. (2012), α-D-glucans exhibit cytotoxic action in relation to human epithelial—HeLa cancer cells. Several *in vivo* studies have shown that polysaccharides exhibit antitumor activity against sarcoma180 in mice (Ferreira et al., 2015). Suarez-Arroyo et al. (2013) used *G. lucidum* as a potential therapeutic agent for inflammatory breast cancer (IBC) using *in vivo* and *in vitro* IBC models. *G. lucidum* has been shown to suppress protein synthesis and tumor growth by affecting signaling pathways for survival and proliferation. A study by Yu et al. (2018) showed that GLPs can activate macrophage cells, increase phagocytosis of macrophages in a dose-dependent manner, and ultimately inhibit the growth of human breast cancer cells. The results of Chen et al. showed that GLPs can stimulate the inflammatory response through the expression of IL -1, IL -6, and TNF-α, which is important for anti-tumor activity (Chen et al., 2004)*.* All these studies prove that the components contained in *Ganoderma* have high antitumor activity.

### Antiviral activity

The results of several studies suggest that *G. lucidum* is a potential candidate for the development of various antiviral agents (Eo et al., 1999). The genomic sequencing indicated that *G. lucidum* could fight against many viruses, such as herpes, influenza, EpsteinBarr, and hepatitis, including the very virulent and dangerous HINI strain of flu.

Lu et al. (2019) investigated the effect of liposomes (Lip) encapsulated with GLP on the inactivation of porcine circovirus type 2, or PCV2. It was found that Lip-GLP can increase the ratio of CD4^+^ to CD8^+^ T cells, enhance the response of PCV2 and stimulate cytokine secretion in mouse serum. Therefore, Lip-GLP is a promising formulation for stimulating immune responses against PCV-II (Liu et al., 2019).

It is known that a dengue virus (DENV) infection leads to serious health problems. The researchers used a computer-generated screening approach to predict triterpenoids from *G. lucidum* as potential inhibitors of DENV NS2B-NS3 protease. Ganodermanotriol was discovered to be the most promising compound against virus infection (Bharadwaj et al., 2019).

Enterovirus 71 (EV71) is a major cause of hand, foot, and mouth disease (HFMD), and lethal neurological and systemic complications in children. Zhang et al. evaluated the antiviral activities of some triterpenoids against enterovirus 71 infection. Some tested triterpenoids have been found to prevent virus infection by blocking virus adsorption to cells (Zhang et al., 2014).

Protein bound polysaccharides were isolated from a water soluble matrix of *G. lucidum* and showed antiviral activities against the herpes simplex virus type 1 (HSV-1) and type 2 (HSV-2) (Eo et al., 2000). Water-soluble and methanol-soluble components from *G. lucidum* showed *in vitro* activities against pathogenic viruses such as herpes simplex virus type 1 (HSV-1) and 2 (HSV-2), influenza A virus (Flu A), and vesicular stomatitis virus (VSV). Some compounds notably inhibited the cytopathic effects of HSV and VSV (Eo et al., 2000).

Some isolated compounds, ganoderic acid beta, lucidumol B, ganodermanondiol, ganodermanontriol, and ganolucidic acid A from *G. lucidum,* have been reported to have inhibitory effects on human immunodeficiency virus (HIV)-1 protease activity (El-Mekkawy et al., 1998; Min et al., 1998). Extensive research is still needed to lay the groundwork for the use of *G. lucidum* isolates as anti-HIV agents. Nevertheless, the triterpenoids appear to be the most important compound with anti-HIV activity (Gao et al., 2003; Cheng et al., 2021).

Ganoderic acid indicates inhibitory effects on the replication of the hepatitis B virus (HBV) (Wachtel-Galor et al., 2011). To date, antiviral potency has been confirmed with several ganoderic acids (GAs) that include GA-A, GA-B, GA-C1, GA-C2, GA-β, GA-T, GA-Q, GA-H, Ganoderol A, Ganoderol B, ganodermanondiol, and ganodermanontriol (Sharma et al., 2019).

The recent outbreak of a new coronavirus (SARS-CoV)-2 is currently a serious global public health threat. Despite the lack of clinical data, there is convincing evidence in the literature that certain nutraceuticals (triterpenoids, polysaccharides, nucleotides, sterols, steroids, fatty acids, and proteins/peptides) found in *G. lucidum* may be useful for the treatment of COVID -19 (Hetland et al., 2021). Results from AL-jumaili et al. (2020) indicate the increase in lymphocytes in *G. lucidum* supplemented to patients with COVID-19. β*-*glucans boost PRRs signals. As a result, protective inflammatory responses are developed that prevent infections by pathogens, including infection with coronaviruses. Therefore, *G. lucidum* can be used to help in the treatment of COVID-19 infections. Triterpene glycosides, which are present in several plants, including *G. lucidum*, inhibited the early stage human coronavirus 229E infection significantly by impairing viral replication, absorption, and penetration (Cheng et al., 2006). *Ganoderma* shows good antiviral potential against DENV and EV71. It also has an inhibitory effect on the protease activity of human immunodeficiency virus (HIV)-1. It is also a promising antiviral agent in COVID -19 infections.

### Anti-inflammatory

A study by Hasnat et al. (2015) demonstrated that *G. lucidum* grown on germinated brown rice showed potential anti-inflammatory effects. Moreover, these results also indicate that GLBR reduces the severity of colitis drastically.

The anti-inflammatory activities of GLPs was determined by carrageenan-induced edema and formalin-induced edema inflammation assays. Results show that polysaccharide inhibited acute inflammation induced by carrageenan and chronic inflammation induced by formalin successfully (Joseph et al., 2011). Similar results were reported by Sheena et al. (2003a), where ethyl acetate and methanol extracts showed significant anti-inflammatory activity in acute and chronic inflammatory models in mice.

Extracts of *G. lucidum* exhibit excellent anti-inflammatory activity due to significant suppression of cytokines: IL -6, IL -23p19, immunomodulatory molecules: S100A7, S100A8, S100A9, and chemokines: CXCL8, CCL5, and CCL20 (Dudhgaonkar et al., 2009). Zhang et al. (2018) investigated the anti-inflammatory properties of sulfated polysaccharides prepared by chemical sulfation. The results showed that the polysaccharide could not only inhibit L-s-electin-mediated inflammation, but also inhibit the whole system.

Su et al. (2020) isolated eight undescribed lanostane triterpenoids from which ganoluciduone B exhibited moderate inhibitory activity on nitric oxide production. All these studies show that extracts of *G. lucidum* have favorable potential as anti-inflammatory agents.

### Neuro-protective activity

Alzheimer’s disease (AD) is a very common chronic progressive neurodegenerative disease. There exists no effective treatment for this illness. Current treatments for Alzheimer’s are focused mainly on improving cognition and relieving symptoms. One of the approaches for treating AD is to control levels of the neurotransmitter acetylcholine in the brain through the inhibition of acetylcholinesterase (AChE). The treatments involve mostly drug therapy, which reduces the symptoms, but is accompanied by many side effects. Alternative treatments involving the use of compounds from *G. lucidum* have been shown to be useful in the treatment of AD (Qin et al., 2019). Zhang et al. (2011) reported that a mixture of triterpenoid compounds stimulates neuronal viability and reduces fatigue. In addition, research has shown that long-term consumption of *G. lucidum* as an adjuvant in the treatment of neurological disorders may reduce the progression of Alzheimer’s disease.

In the study by Rahman et al. (2020) rats were tested for memory and learning for six consecutive days. Rats fed with *G. lucidum* water extract needed less time to search items and used the shorter route. This means that their spatial perception and memory improved. Authors also reported the inhibition of acetylcholinesterase (AChE) of *G. lucidum* extracts from 22.5% to 50% (Hasnat et al., 2013; Cör et al., 2014). Lai et al. (2019) described that those alcoholic extracts from *G. lucidum* affect DNA methyltransferases by regulating DNA methylation. This may be an important signaling pathway influenced by *G. lucidum* in inhibiting the progression of AD. This extract, including ganoderic acid and lucidone A, may contribute to inhibiting the progression of AD. Aromatic compounds, including meroterpenoids and alkaloids, were investigated for their neuroprotective activity against CORT -induced PC12 cell damage. Their structures were determined by spectroscopic methods. The compounds showed remarkable neuroprotective activities against corticosterone-induced PC12 cell damage, and exhibited significant anti-inflammatory activities against LPS-induced nitric oxide production (NO) in RAW264.7 macrophages (Lu et al., 2019). From this point of view, *G. lucidum* appears to be promising in preventing the pathogenesis of Alzheimer’s disease caused by hypercholesterolemia.

## Methods for extracting biologically active substances from *G. lucidum*

As mentioned above, a number of polysaccharides have been extracted and isolated from *Ganoderma* that have different chemical structures and have been shown to possess bioactive properties. The choice of extraction method for polysaccharide extraction is very important. Depending on the type and water solubility of the polysaccharide, the method of extraction is chosen. Usually, the cell wall is split from the outer to the inner part. Most polysaccharides can be extracted with hot water, acid saline, dilute alkaline solutions and dimethyl sulfoxide (Nie et al., 2013).

The most commonly used method for extracting polysaccharides from Ganoderma is hot water extraction. High temperatures are required to accelerate the dissolution of polysaccharides from cell walls. Usual extraction procedures are carried out by first defatting the base solids with organic solvents. This removes low molecular weight substances. Extraction is then carried out with hot water, saline solution and dilute alkaline solutions at different temperatures. Extraction with hot water is usually carried out for 3 h at 100°C. Hot water extraction is usually carried out at 100°C for 3 h, while 2% ammonium oxalate at 100°C for 6 h and 5% sodium hydroxide at 80°C for 6 h are used for extraction with saline and dilute alkaline solutions, respectively (Wang et al., 2011). Extraction with hot water yields water-soluble polysaccharides, while extraction with alkali yields water-insoluble polysaccharides (Nie et al., 2013). In addition to conventional extraction, various extraction methods such as microwave, ultrasonic, ultrasonic/microwave and enzyme extraction are also used for polysaccharide extraction, which can accelerate cell wall disruption and increase the yield of polysaccharides (Xu et al., 2007; Huang and Ning, 2010; Zhao et al., 2010; Ma et al., 2013; Kang et al., 2019). After extraction, the free proteins must be removed. The Sevag method is commonly used, in which the protein is precipitated after repeated denaturation by shaking with a solution of octanol in chloroform (Staub, 1965). To obtain crude polysaccharides the deproteinized solution is then precipitated with alcohol, methanol or acetone.

Since *G. lucidum* neutraceuticals are in high demand, the challenge is to increase the amount of bioactive compounds. The amount of active components in the mushroom is certainly influenced by the cultivation conditions (temperature, aeration, light, pH, humidity, growth media etc.) In the study by Sudheer et al. (2016b) the effect of ozone as an elicitor on the increase of bioactive compounds in the fungus is investigated. It was shown that the application of ozone doubles the bioactive compounds in the fungus and also increases the antioxidant activity of the extracts. Aeration and light also affect the growth and amount of bioactive compounds in the fungus. CO_2_ concentrations between 2%–5% stimulated the growth of antler-like fruiting bodies. Fungi growing under these conditions also showed higher biological activity (Sudheer et al., 2018). The growth medium is important for the development of the bioactive components of the fungus. Researchers also report the cultivation of *G. lucidum* on various types of sawdust, harrows, and especially on various agricultural wastes (Peksen and Yakupoglu, 2009; Gurung et al., 2012; Khoo et al., 2022). In one of the research been shown that the waste biomass of oil palm fibre the main agricurtural waste in Malaysia can be a good substrate for the growth of *G. lucidum*. Parameters such as temperature, pH, humidity, and the carbon and nitrogen composition required for optimal mycelial growth were studied and determined (Sudheer et al., 2016a). Chromatographic techniques such as ion exchange chromatography, gel filtration chromatography and affinity chromatography are most commonly used to obtain pure polysaccharides (Jiang et al., 2012).

The lanostane-type triterpenoids isolated from *G. lucidum* can be categorized as *Ganoderma* alcohols because of the hydroxyl group on the lanostane part or as *Ganoderma* acids because of the carboxyl group on the side chain. Triterpenoids can be extracted from *Ganoderma* with organic solvents or aqueous solutions. Pure triterpenoids are obtained with organic solvents, while aqueous mixtures yield triterpenoids that may also contain polysaccharides. Various methods are used to extract triterpenoids, such as microwave extraction, ultrasonic extraction, Soxhlet extraction, solvent extraction at elevated temperature etc. (Chen et al., 2007; Taofiq et al., 2017; Oludemi et al., 2018). The aim is to break the cell walls and gain access to the pharmacological active ingredients. However, extraction with hazardous organic solvents is harmful. For this reason, there is a growing trend toward the use of environmentally friendly technologies that avoid the use of hazardous organic solvents. One such technology is supercritical extraction. Dat et al. (2022) used supercritical CO_2_ to extract triterpenoids from *Ganoderma*. They demonstrated the cytotoxic effect of *G. lucidum* extract on cancer cells kb, HepG2, Lu, and MCF7 which indicate the potential of *G. lucidum* extract as an anticancer agent. Extensive laboratory and preclinical studies show that several purified triterpenes or triterpene-containing extracts of *G. lucidum* have a direct anticancer effect. For this reason, it is very important what technique is used, and in particular, what type of solvent is used to obtain a particular component. Therefore, it is necessary to consider the extraction of triterpenoids by more environmentally friendly extraction methods. Therefore, SC-CO_2_ extraction could be considered as an alternative and important method for the production of high quality compounds. Analytical methods for the characterisation and quantification of triterpenoids include: gas chromatography, liquid chromatography, thin-layer chromatography, supercritical fluid chromatography, NMR spectroscopy, capillary electrophoresis, and X-ray spectroscopy (Xu et al., 2018).

## Discussion and future aspects

From the numerous studies that have been conducted and published, it can be summarised that *G. lucidum* may have various beneficial therapeutic properties. Extracts of *Ganoderma* have been reported to have antioxidant (Cherian et al., 2009; XiaoPing et al., 2009; Fan et al., 2012; Dong et al., 2019; Jeong and Park, 2020), antibacterial (Kalam et al., 2010; Heleno et al., 2013; Shah et al., 2014; Mishra et al., 2018; Sande et al., 2020), antifungal (Wang and Ng, 2006; Yang et al., 2022), antitumor (Sun et al., 2011; Li et al., 2013; Yu et al., 2018; Jiao et al., 2020; Shahid et al., 2022), antiviral (Eo et al., 2000; Wachtel-Galor et al., 2011; Zhang et al., 2014; AL-jumaili et al., 2020; Hetland et al., 2021), antiimflamatory activities, among others. These properties are attributed to numerous bioactive compounds such as triterpenes, polysaccharides, proteins and others.

Chemopreventive and therapeutic studies have shown how certain plant extracts act on various types of diseases, including cancer. *G. lucidum* is a very popular edible mushroom. It has been shown in numerous studies to modulate a number of signaling pathways responsible for the abnormal characteristics of cancer cells. Its effects are manifested in cell cycle slowing, induction of apoptosis, growth inhibition, and suppression of metastatic behaviour. Its action is shown by the inhibition of the constitutively active transcription factors NF-κB and AP -1, which have been mentioned as potential therapeutic targets for cancer therapy. Inhibition of NF-kb is extremely important for cancer prevention and treatment. Its function is to control the expression of proteins involved in cell adhesion, migration, and invasion (uPA, uPAR) (Jiang et al., 2008; Chen et al., 2010), proteins that protect against cell death (Bcl-2, BclXL) (Hsu et al., 2008), of oncoproteins that promote cell cycle dysregulation and tumorigenesis (cyclin D1) (Shahid et al., 2022), and of angiogenic factors that promote tumour growth (Hsu et al., 2009).

*G. lucidum*, in conjunction with its phenolic compounds, triterpenes, polysaccharides, and peptides, exhibits high antioxidant biological activity (Jiang et al., 2012; Kao et al., 2012; Dong et al., 2019; Jeong and Park, 2020; Zheng et al., 2020). Consumption of antioxidant-enriched foods has been shown to protect against cancer and other chronic diseases (Abdullah et al., 2012). However, this has not been explicitly demonstrated for antioxidants from *G. lucidum*. Therefore, further studies are needed to clarify the effect of antioxidants from *Ganoderma* on the host immune system. *G. lucidum* has also been described as a promising source of antimicrobial molecules, especially polysaccharides, against various viral (AL-jumaili et al., 2020; Cheng et al., 2021), bacterial (Radhika, 2021), and fungal pathogens (Wang and Ng, 2006; Yang et al., 2022). Scientific studies investigating antiviral activity have been conducted mainly on animals. Zhu et al. (2017) studied the anti-influenza effect of a hot aqueous extract of *G. lucidum* in infected mice, and the effect was very limited. Therefore, further studies are needed to confirm and improve the functional use of this fungus against influenza. The NS2B-NS3pro of dengue virus was recently identified as an ideal target for the development of new anti-dengue drugs. Ganodermanontriol from *G. lucidum*, a potent bioactive triterpenoid, has been reported to inhibit DENV-NS3pro based on *in vitro* studies. With further studies, ganodermanontriol could act as a drug against DENV virus (Bharadwaj et al., 2019). Furthermore, lanosta-7,9(11),24-trien-3-one, 15; 26-dihydroxy and ganoderic acid Y were also described as active against EV71 infections. Both compounds were shown to significantly inhibit viral RNA replication (vRNA) of EV71 by blocking EV71 uncoating. Therefore, both could be two promising agents to control and treat EV71 infections (Zhang et al., 2014). There is also an idea of using *Ganoderma* in the treatment of the new coronavirus. β-Glucans enhance PRR signaling. This develops protective inflammatory responses that prevent infections by pathogens, including infections by coronaviruses. Therefore, after further studies, *G. lucidum* can be used in the treatment of COVID -19 infections (AL-jumaili et al., 2020). In the field of neuroprotective effect. There are also studies that confirm that *Ganoderma* can have a positive influence on the development of Alzheimer’s disease (Zhang et al., 2011).

Much of the research on *G. lucidum* reports positive clinical results and potential therapeutic applications. For all this reason, *G. lucidum* and its derived products are still widely used as commercial products. However, despite its great importance, both the efficacy and safety of *G. lucidum* consumption have been insufficiently studied. Its safety and potential toxicity to humans have been poorly studied. One study reported that *G. lucidum* extracts may be toxic *in vitro* (Hapuarachchi et al., 2018). However, treatment with powdered *G. lucidum* spores even caused hepatotoxic effects (Wanmuang et al., 2007). Given the popularity of the mushroom, there is an urgent need for further research to gain a comprehensive understanding of its biomechanisms in addition to the numerous *in vitro* and *in vivo* studies already reported. And consequently, their biotherapeutic applicability. The methods used to isolate the active compounds from *Ganoderma* should also be considered. The polysaccharides are extracted mainly with hot water and the triterpenoids with organic solvents. However, it is obviously undesirable to extract the extracts with hazardous organic solvents. Therefore, scCO_2_ extraction is a promising method to obtain these compounds. To decipher the bioactive potency of these compounds, their proper isolation, purification, and identification is crucial. Further research is required to study the bioactive constituents of *G. lucidum* in detail. This will be useful for further use of these constituents in clinical trials and confirm or refute some of the study results.

## Conclusion

The antioxidant, antimicrobial, antifungal, antitumor, antiviral, anti-inflammatory, and nevro-protective activities of the isolated compounds from *G. lucidum* were summarized in the present review. To date, studies on the bioactive activities of *G. lucidum* have focused on two groups of chemicals: triterpenoids and polysaccharides. Of the triterpenoids, ganoderic acids have been shown to be cytotoxic to a variety of cancer cell lines (breast, lung, liver, colon, etc.) and are therefore thought to be responsible for the antitumor activity. In addition to ganoderic acids, GLPs are also associated with antitumor activity, because they exhibit immunomodulatory activities. The properties of polysaccharides are probably related mainly to the molecular weight, degree of branching, and water solubility of polysaccharides. In the last three decades several GLPs have been extracted by different methods according to their structure. The most commonly isolated polysaccharides from *G. lucidum* are α- or β-(1→3)-, (1→6)-glucans and heteropolysaccharides with different sugar combinations.

A comprehensive understanding of the nutritional and therapeutic roles of G. lucidum extracts is essential for the development of new drugs and various functional foods. Further studies on the isolated compounds of *G. lucidum* are still needed, focusing on specific components of the bioactive compounds. These characterized components need to be evaluated by *in vitro* and *in vivo* studies to determine the exact amounts for further clinical studies. Since isolated compounds from *G. lucidum* have also been shown to be antiviral and immunomodulatory agents, this may also be a promising potential source for the current SARS-CoV-2 pandemic. Newly gained knowledge and further studies could facilitate the development of new nutraceuticals and pharmacological formulations from *G. lucidum*.

## Abbreviations

CAT, catalase; CCL20, chemokine (C-C motif) ligand 20; CCL5, chemokine (C-C motif) ligand 5; CXCL8, C-X-C motif chemokine ligand 8; G-CSF, granulocyte colony stimulating factor; GM-CSF, myelopoietic growth factor and pro-inflammatory cytokine; GPX, glutathione peroxidase; IFN-γ, interferon-gamma; IL-1β, interleukin 1 beta; IL-6, interleukin 6; MAPK, mitogen-activated protein kinase; M-CSF, macrophage colony-stimulating factor; MDA-MB-231, human breast cancer cells; MT-1, of human breast malignant carcinoma cells; NM23-H1, Non-metastatic protein 23 H1; PKC, Protein kinase C; PRRs, pattern recognition receptors; S100A7, S100 calcium binding protein A7; S100A8, S100 calcium binding protein A8; S100A9, S100 calcium-binding protein A9; SMase, sphingomyelinase; SOD, superoxide dismutase; T cell, T lymphocyte; TNF-α, tumor necrosis factor alpha.
